# Patient-initiated brief admission: a single site eight-year retrospective cohort study

**DOI:** 10.1017/neu.2025.10031

**Published:** 2025-08-26

**Authors:** Melker Hagsäter, Monica Ohlsson, Meritxell Casanovas Roca, Axel Sjöstedt, Fredrik Hieronymus

**Affiliations:** 1 Institute of Neuroscience and Physiology, Department of Pharmacology, the Sahlgrenska Academy at the University of Gothenburg, Gothenburg, Sweden; 2 Kungälv Hospital, Västra Götaland Regional Council, Kungälv, Sweden

**Keywords:** Crisis intervention, hospitalization, patient admission, patient participation, voluntary admission

## Abstract

**Objective:**

Patient-Initiated Brief Admission (PIBA) is an intervention designed to provide constructive crisis management for patients. The purpose of this study was to evaluate outcomes in healthcare utilisation and self-inflicted injuries at one Swedish Hospital where PIBA was implemented in late 2017.

**Methods:**

Patients who signed a PIBA-contract between 2017 and 2023 were included in the study. Data on inpatient care, contacts with the psychiatric emergency department and self-inflicted injuries that resulted in contact with medical care were collected from patients’ medical records. Effects of PIBA were assessed using paired *t*-tests, comparing pre-post changes 0.5, 1 and 2 years, from initiation of PIBA-contract, respectively.

**Results:**

Data from a total of 38 patients were analysed. There was a marked decrease in inpatient care from voluntary admissions in the first six months after initiation of PIBA. There was also a significant decrease in number of contacts with the psychiatric emergency department (for all patients) in the 1-year pre-post comparison, but not for the 0.5- and 2-year pre-post comparisons. There were no significant reductions in compulsory inpatient care or self-inflicted injuries in our cohort. Patients with contracts extending over several years appeared stable, on average, in their use of care and prevalence of intoxications.

**Conclusion:**

The main effect on inpatient care after initiation of PIBA was a reduction in voluntary admissions, coinciding with a shift from voluntary admissions in favour of PIBA. The results support a more widespread utilisation of PIBA from a health-economic perspective.

Significant outcomes

- Patient-initiated brief admission reduced voluntary admissions.
- Patient-initiated brief admissions did not reduce self-inflicted injuries.
- Patient-initiated brief admission is likely beneficial from a health-economic perspective.

Limitations

- The study employs a retrospective and naturalistic design.
- The study lacks a control group.

## Introduction

Patient-initiated brief admission (PIBA), is an intervention which gives eligible patients a level of control over admission to inpatient care (Eckerstrom *et al*., 2022; Moberg & Schon, 2022; Eckerstrom *et al*., 2024; Lindgren *et al*., 2024; Hultsjo *et al*., 2025; Varna *et al*., 2025). While PIBA is the preferred acronym in recent publications, several other acronyms have also been used to refer to the model, including brief admission (BA), patient-controlled admission (PCA), self-admission (SA), self-controlled admission (SCA), and self-referral to inpatient treatment (SRIT) (Heskestad & Tytlandsvik, 2008; Strand & von Hausswolff-Juhlin, 2015; Thomsen *et al*., 2018; Eckerstrom *et al*., 2019; Westling *et al*., 2019; Strand *et al*., 2020; Skott *et al*., 2021). The care structure of PIBA includes defined parameters regarding the length, content, and purpose of the care, as well as patient obligations – such as refraining from self-harm during inpatient treatment. The conditions are stipulated in a non-binding contract signed by both the patient and the caregiver. Previous studies on PIBA have primarily been conducted in Sweden, Norway, Denmark, the Netherlands and Australia; for a recent review, see Värnå et al., (Varna *et al*., 2025). PIBA has been applied for a broad spectrum of psychiatric conditions, including complex comorbidities, with the bulk of the evidence stemming from patients with schizophrenia (Moljord *et al*., 2016; Thomsen *et al*., 2018; Skott *et al*., 2021), bipolar disorder (Helleman *et al*., 2014b), anorexia nervosa (Strand *et al*., 2020) and emotional instability (Koekkoek *et al*., 2010; Eckerstrom *et al*., 2019; Westling *et al*., 2019).

Studies assessing patients’ experience of self-referral are generally positive, emphasising that inpatient care through PIBA provides them comfort and a sense of security, temporary retreat from problems in the outside world and fosters their self-growth and responsibility (Strand & von Hausswolff-Juhlin, 2015; Varna *et al*., 2025). It has also been proposed that involving patients in decision making could result in a reduced need for inpatient care and coercive interventions (Helleman *et al*., 2014a; Strand & von Hausswolff-Juhlin, 2015; Moljord *et al*., 2016). PIBA has been reported to reduce certain outcomes – such as compulsory admissions, self-injuries (Westling *et al*., 2019), as well as total inpatient care (Nyttingnes & Ruud, 2020; Strand *et al*., 2020; Skott *et al*., 2021). However, most studies that include a control group suggest that the model does not decrease total inpatient care compared to the control (Sigrunarson *et al*., 2017; Thomsen *et al*., 2018; Westling *et al*., 2019; Eckerstrom *et al*., 2024).

The aim of this retrospective cohort study was to examine the impact of the introduction of PIBA on psychiatric care consumption at Kungälv Hospital, as well as its effects on potentially related outcomes. It was the first study performed on this cohort. The measures that were analysed, through pre-post comparisons, were inpatient care (total and divided into PIBA, voluntary and compulsory), contacts with the psychiatric emergency department, and self-inflicted injuries (total and divided into subtypes) resulting in contact with the medical care.

## Methods

### Study design

The study was designed as a retrospective cohort study. Data were collected from the patients’ medical records (by manual review) in the interval from two years prior to initiation of PIBA-contract, that is, November 30, 2015 (for the first patient) until December 31, 2023 (all patients remaining in the catchment area). Information about inpatient care (total and divided into PIBA, voluntary and compulsory), contacts with the psychiatric emergency department, self-inflicted injuries (divided into self-harm, intoxication and strangulation) that resulted in contact with the medical care and demographic data, such as information about age, sex and diagnoses given to the patients during the six months prior to signing a PIBA-contract, were collected. Self-harm included cuts, burns, swallowing sharp objects or batteries, and self-embedding. The medical care included contacts with the medical emergency department, the intensive care unit and, while on inpatient care, medical consultant contacts resulting in active interventions, such as stitching self-inflicted wounds.

### Intervention and participants

Kungälv Hospital, Sweden, with a catchment area of 140 000 inhabitants, implemented PIBA in late 2017. The implementation of PIBA at Kungälv Hospital was based on the core components of BA as described by Helleman et al., (Helleman *et al*., 2014a; Helleman *et al*., 2014b; Westling *et al*., 2019). The implementation was primarily intended for, but not limited to, patients with self-injury and risk of suicide. However, eligibility was ultimately based on a clinical judgment that the patient was deemed likely to benefit from the model. The PIBA-contract allowed for three inpatient visits per month, each with a maximum duration of three nights. Self-referring patients must contact the psychiatric ward between 8 AM and 8 PM, after 8 PM the two single rooms reserved for PIBA could be used for other patients. The patient is admitted by a registered nurse. During inpatient care the patient is allowed to participate in activities arranged at the ward. The patient can, if agreed on in the contract, be offered one or two daily conversations with nursing staff, each lasting 15–20 minutes, but the patient is not allowed consultations with a psychiatrist or a psychologist while on PIBA. Neither are changes in prescription drugs to be discussed during PIBA. The patient must refrain from self-injury, and otherwise comply with the rules at the ward. The need for PIBA should be evaluated once a year and – if found beneficial to the patient – the PIBA-contract should be extended.

### Outcome measures

The primary outcome measures were:

1. Total number of inpatient care days, with the following sub-analyses:

a) excluding PIBA admissions

b) excluding compulsory care

c) including only compulsory care

d) including only voluntary care

2. Number of contacts with the psychiatric emergency department

3. Number of medical care contacts due to self-inflicted injury, with the following sub-analyses:

a) including only intoxications

b) including only self-harm

c) including only strangulations

4. Total utilisation of PIBA

### Sensitivity analyses

When working with the material, it became apparent that a handful of participants were disproportionately represented with respect to certain self-harm events. To ascertain that no underlying trend was missed due to extreme outliers, two sensitivity analyses were performed: (1) one excluding three patients who together accounted for more than 60 % of the total number of contacts with medical care resulting from self-inflicted injuries and (2) excluding one patient who represented 35 % of all medical care contacts following intoxication.

### Statistical analysis

Analyses were performed in R version 4.3.1. Both the intention-to-treat sample (all patients that were included in the model) and the in-treatment sample (patients that remained in the model) were analysed. Treatment effects were assessed with paired t-tests comparing pre-post changes 0.5, 1 and 2 years (e.g. the number of inpatient care days two years prior to initiation of PIBA compared to two years after), respectively. A two-sided p-value of 0.05 was considered statistically significant. Due to the exploratory nature of the study and the high degree of intercorrelation between outcome measures (overlapping both in terms of time and type), no correction for multiple testing was performed and *p*-values should be interpreted descriptively. All patients that were included in the model were eligible for analyses, but patients were only included in those analyses where they could have data for the full analysis interval. For example, a patient residing in Kungälv for 1.5 years prior to PIBA-initiation, and/or having exactly 1.5 years of follow-up, would be included in the ± 0.5 and ± 1-year analyses, but not in the ± 2-year analysis. When visually presenting the data, all patients with complete data for the corresponding interval are included, for example, a patient with complete data from the first year after PIBA-initiation, but who lacks complete data from the second year after PIBA-initiation, is displayed in the 1-year column but not the 2-year column.

## Results

### Study population

Demographics of the study cohort are presented in Table 1. The mean age at inclusion in the model was 34 years (SD = 12.1). Patients were predominantly female (84 %), and the most common given diagnoses were mood disorders (74 %) and specific personality disorders (61 %). One patient established contact with the hospital’s psychiatric unit less than six months prior to signing a PIBA-contract (four months). For that patient, demographic data was extracted for the available period only.

**Table 1.** Demographics

		*n*	%
Age at inclusion	Below 20 20 – 29 30 – 39 40 – 49 50 – 59 60 – 69	3 14 10 7 3 1	8 37 26 18 8 3
Gender	Female Male Transgender	32 4 2	84 11 5
Psychiatric inpatient care 0 – 6 months prior to PIBA-contract	Any Voluntary Compulsory	33 33 7	87 87 18
Contact(s) with the psychiatric emergency department 0 – 6 months prior to PIBA-contract	Yes No	20 18	53 47
Medical care due to self-inflicted injuries 0 – 6 months prior to PIBA-contract	Any Intoxication Self-harm Strangulation	14 11 6 1	37 29 16 3
Diagnoses (ICD 10) 0 – 6 months prior to PIBA-contract	Schizophrenia, schizotypal and delusional disorders (F20–29) Bipolar affective disorder (F30–31) Mood [affective] disorders other than Bipolar affective disorder (F32–39) Specific personality disorders (F60) and problems related to life-management difficulty (Z73) Gender identity disorders (F64) Mental retardation (F70–79) Hyperkinetic disorders (F90)	2 9 28 23 1 4 2	5 24 74 61 3 11 5

*Patient characteristics at time of PIBA-inclusion or during 0 – 6 months prior to inclusion.*

### PIBA utilisation and reasons for termination of contract

A total of 38 patients signed a PIBA-contract between November 2017 and December 2023 (Table 2). The number of patients who were offered PIBA but declined was not available. A total of 16 patients (42 %) remained in the programme by the end of 2023. The reasons for cessation of contract were expiration without renewal of contract not otherwise specified (*n* = 11), patient moved outside of catchment area (*n* = 6), termination by caregiver due to patient being unable to manage PIBA (*n* = 3) and termination on patient’s initiative (*n* = 2). The occupancy rate of the two single rooms reserved for PIBA, as used for PIBA, was between 15 % and 19 % per year (Table 2).

**Table 2.** Utilisation of PIBA and changes in number of patients with PIBA-contract per year

	2017	2018	2019	2020	2021	2022	2023
No. patients with contract (by end of year)	1	13	10	14	17	13	16
No. patients that used PIBA at least once during the year	1	11	10	12	11	11	7
No. new contracts	1	13	3	6	5	3	7
No. ended contracts	0	1	6	2	2	7	4
Occupancy rate (%)	–	15	19	17	15	18	18

There were marked inter-individual differences in PIBA use. Among patients with full data coverage in the first six months after PIBA-initiation (*n* = 34), five patients accounted for more than half of the total PIBA utilisation (17 %, 12 %, 10 %, 8 %, and 8 %, respectively), while 19 patients used PIBA a total of three days or less. Initial usage was also indicative for future use, for example, none of the patients who used PIBA a total of three days or less in the first six months after PIBA-initiation used PIBA for more than three days in any subsequent six-month interval (Supplementary S1).

### Effect of PIBA on inpatient care

**Figure 1.**
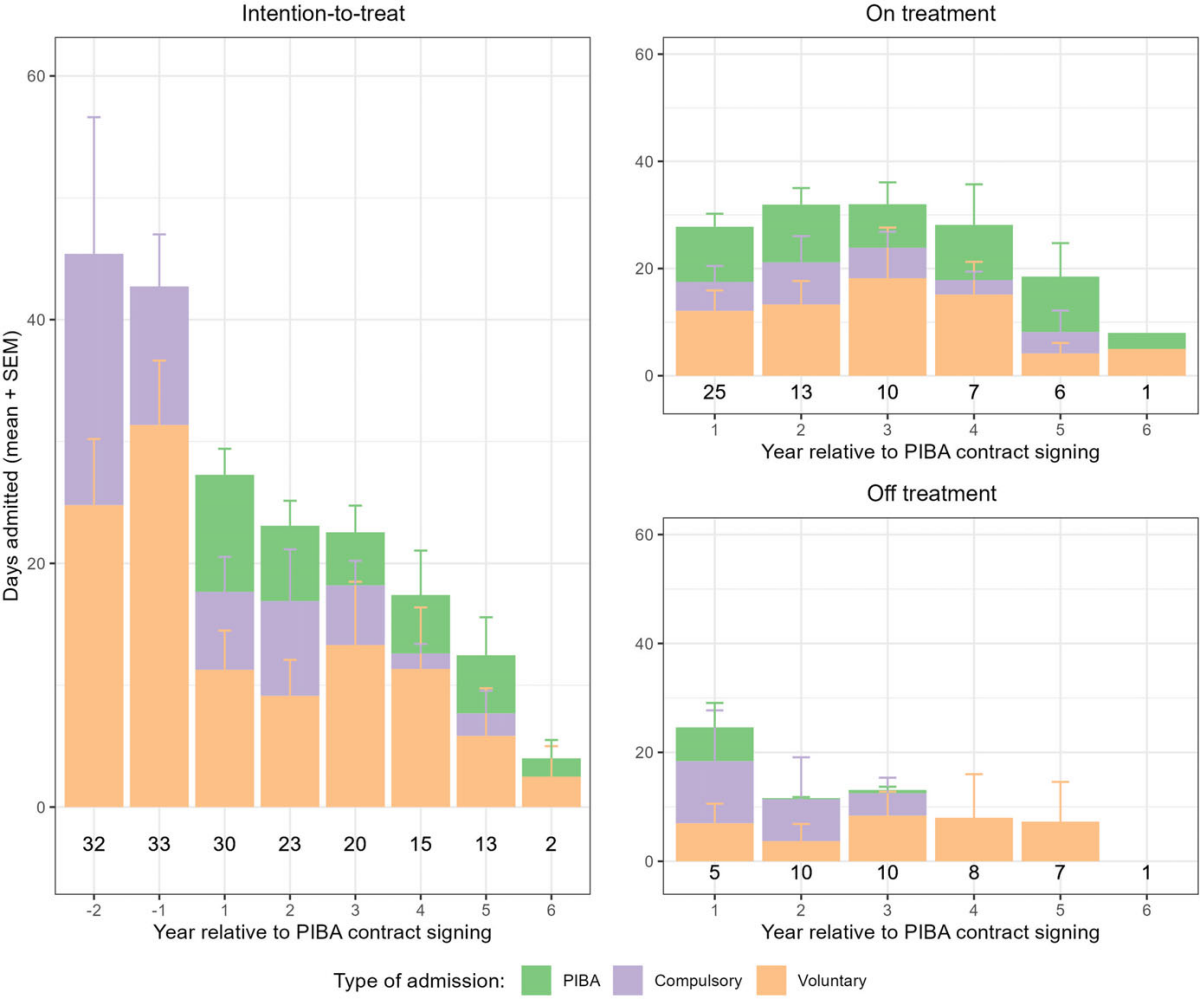
Days admitted to inpatient care, stratified by type of admission. Inpatient care displayed as average days per year. Numbers displayed below the bars are the number of patients with full data coverage in respective interval (year relative to PIBA-contract signing). Patients with only partial data in respective interval are not displayed. The left panel (year -2 to 6) shows inpatient care for all patients who signed a PIBA-contract. Top right panel displays patients with ongoing PIBA-contract in the respective interval (year 1 to 6) and the bottom right panel (year 1 to 6) displays patients with ceased PIBA-contract within respective, or a former interval.

The total use of psychiatric inpatient care was numerically lower after initiation of PIBA for all subsequent years compared to the two years before PIBA (Fig. 1). For the analysed intervals, there was a significant difference for the ± 2-year interval [*t*_±2_(21) = −2.319, *p* = .031], and near significant differences for the ± 0.5- and ± 1-year intervals [*t*_±0.5_(32) = −1.972, *p* = .057; *t*_±1_(27) = −1.807, *p* = .082]. There were no significant effects for comparisons of patients with ongoing PIBA-contracts only (in-treatment) [*t*_±0.5_(32) = −1.972, *p* = .057; t_±1_ (22) = −1.760, *p* = .092; *t*_±2_(11) = −0.820, *p* = .429].

Total inpatient care excluding PIBA was decreased for all examined intervals both for all patients [*t*_±-0.5_(32) = −3.819, *p* = .001; *t*_±1_(27) = −3.454, *p* = .002; *t*_±2_(21) = −3.291, *p* = .003] and for patients with ongoing PIBA-contracts only [*t*_±0.5_(32) = −3.819, *p* = .001; *t*_±1_(22) = −3.292, *p* = .003; *t*_±2_(11) = −3.035, *p* = .011].

There were no significant pre-post differences in number of days in compulsory care for all patients [*t*_±0.5_(32) = −1.244, *p* = .223; *t*_±1_(27) = −0.427, *p* = .673; *t*_±2_(21) = −1.389, *p* = .179] nor for patients with ongoing PIBA-contracts only [*t*_±0.5_(32) = −1.244, *p* = .223; *t*_±1_(22) = −0.885, *p* = .386; *t*_±2_(11) = −0.473, *p* = .645].

Number of days in voluntary care decreased significantly for all examined intervals, both for all patients [*t*_±0.5_(32) = −4.395, *p* < .001; *t*_±1_(27) = −4.637, *p* < .001; *t*_±2_(21) = −5.158, *p* < .001] and for patients with ongoing PIBA-contracts only [*t*_±0.5_(32) = −4.395, *p* < .001; *t*_±1_(22) = −4.083, *p* < .001; *t*_±2_(11) = −3.418, *p* = .006].

Number of days in voluntary care and PIBA combined decreased significantly in the ± 1- and ± 2-year intervals for all patients [*t*_±0.5_(32) = −1.327, *p* = .194; *t*_±1_(27) = −2.181, *p* = .038; *t*_±2_(21) = −2.675, *p* = .014], but not for patients with ongoing PIBA-contracts only [*t*_±0.5_(32) = −1.327, *p* = .194; *t*_±1_(22) = −1.786, *p* = .087; *t*_±2_(11) = −0.816, *p* = .432].

### Effect of PIBA on contacts with the psychiatric emergency department

**Figure 2.**
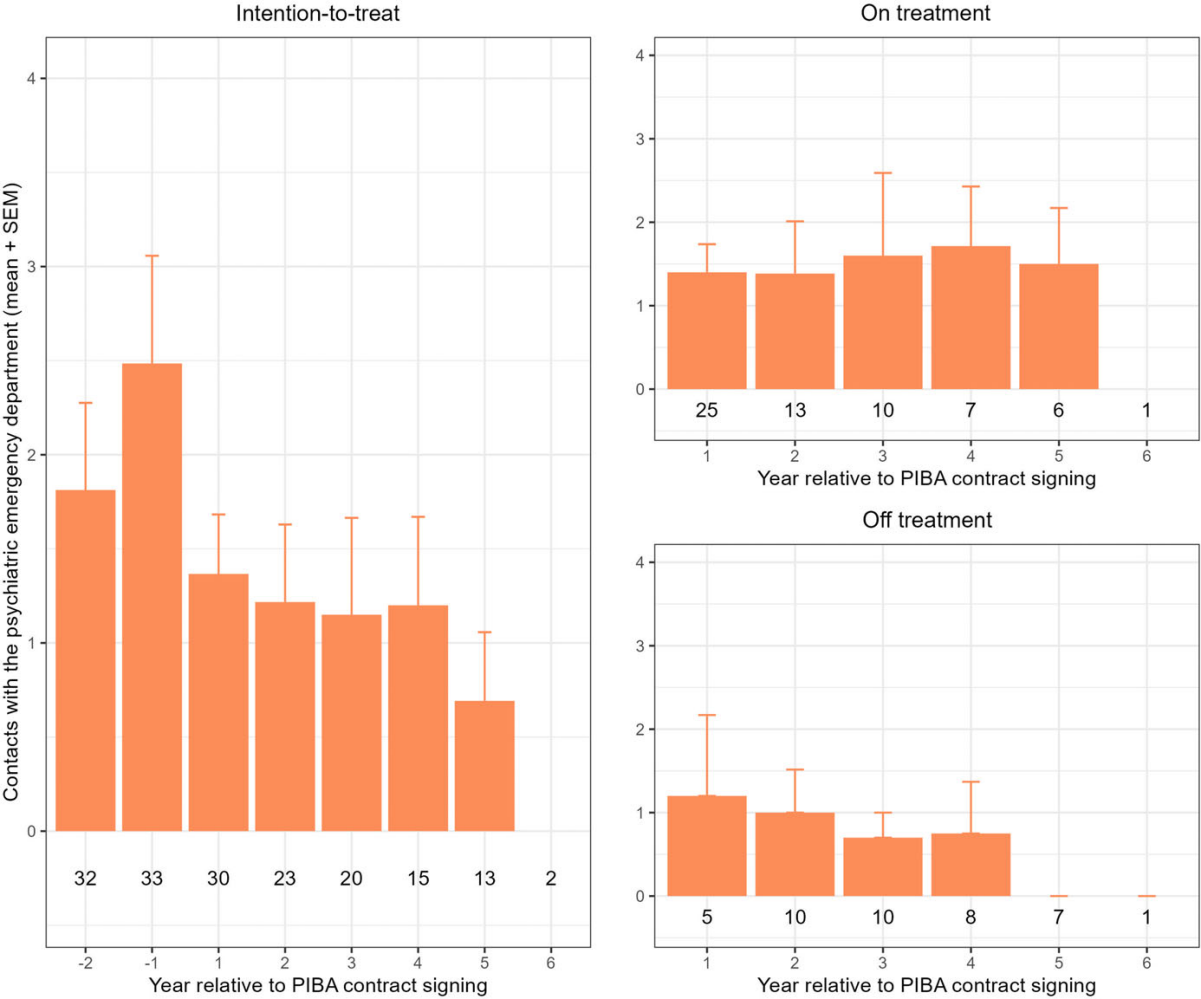
Contacts with the psychiatric emergency department. Contacts with the psychiatric emergency department displayed as average number of contacts per year. Numbers displayed below the bars are the number of patients with full data coverage in respective interval (year relative to PIBA-contract signing). Patients with only partial data in respective interval are not displayed. Left panel (year -2 to 6) shows inpatient care for all patients who signed a PIBA-contract. Top right panel (year 1 to 6) displays patients with ongoing PIBA-contract in respective interval. Bottom right panel (year 1 to 6) displays patients with ceased PIBA-contract within respective, or a former interval.

The average number of contacts with the psychiatric emergency department was lower (all years) after initiation of PIBA compared to the two years before contract (Fig. 2). The total number of contacts with the psychiatric emergency department was lower for the group with ceased PIBA-contracts compared to the patients with ongoing PIBA-contracts for all years (1 – 6). Pre-post comparisons of all eligible patients displayed a significant decrease in the ± 1-year interval [*t*_±1_(27) = −2.126, *p* = .043] but not in the ± 0.5- and ± 2-year intervals [*t*_±0.5_(32) = −0.466, *p* = .645; *t*_±2_(21) = −1.699, *p* = .104], respectively. The effect seen for the ± 1-year interval remained significant when including only patients with ongoing PIBA-contracts [*t*_±0.5_(32) = −0.466, *p* = .645; *t*_±1_(22) = −2.079, *p* = .049; *t*_±2_(11) = −1,477, *p* = .168].

### Effect of PIBA on self-inflicted injuries resulting in contact with the medical care

**Figure 3.**
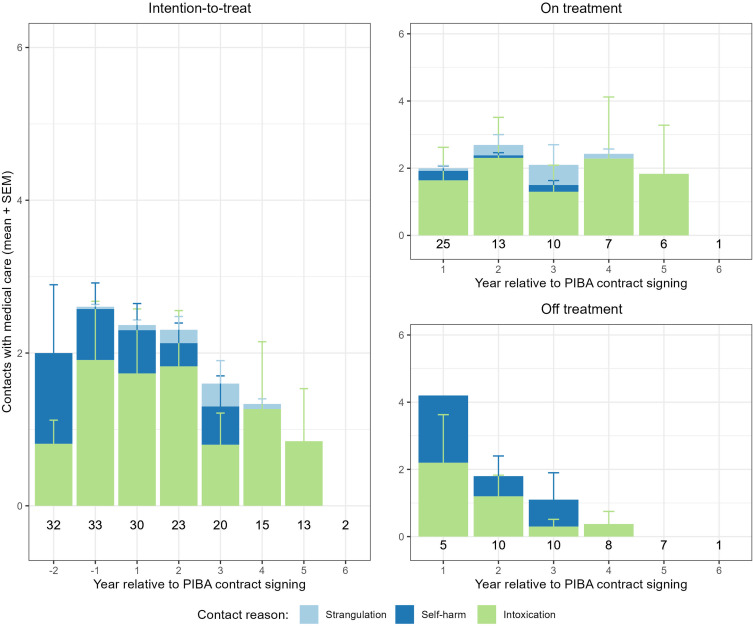
Contacts with medical care due to self-inflicted injuries, stratified by type of injury. Self-inflicted injuries resulting in contacts with the medical care displayed as average number of contacts per year. Numbers displayed below the bars are the number of patients with full data coverage in respective interval (year relative to PIBA-contract signing). Patients with only partial data in respective interval are not displayed. Left panel (year -2 to 6) shows inpatient care for all patients who signed a PIBA-contract. Top right panel (year 1 to 6) displays patients with ongoing PIBA-contract in respective interval. Bottom right panel (year 1 to 6) displays patients with ceased PIBA-contract within respective, or a former interval.

For all eligible patients, there were no significant pre-post differences in total number of contacts with medical care due to self-inflicted injuries (all types) [*t*_±0.5_(32) = 0.138, *p* = .891; *t*_±1_(27) = −0.580, *p* = .567; *t*_±2_(21) = −0.641, *p* = .528] (Fig. 3). Neither were there any significant pre-post differences for patients with ongoing PIBA-contracts only [*t*_±0.5_(32) = 0.138, *p* = .891; *t*_±1_(22) = −1.515, *p* = .144; *t*_±2_(11) = 0.225, *p* = .826]. Over time, the total number of contacts with medical care (all types) was numerically lower in the group with ceased PIBA-contracts compared to the group with ongoing PIBA-contracts. A sensitivity-analysis, excluding three patients who together were responsible for more than 60 % (25 %, 23 %, and 13 %, respectively) of the total number of contacts with medical care due to self-inflicted injuries in the ± 2-year interval (all available data), did not reveal any significant effects (intention-to-treat: [*t*_±0.5_(29) = −1.756, *p* = .090; *t*_±1_(24) = −0.816, *p* = .422; *t*_±2_(18) = 0.256 *p* = .801], in-treatment: [*t*_±0.5_(29) = −1.756, *p* = .090; *t*_±1_(20) = −1.234, *p* = .232; *t*_±2_(9) = 1.272, *p* = .235]) (Supplementary S2).

Sub-analyses were performed by type of self-inflicted injury. Only one patient in the cohort had strangulation events resulting in contacts with the medical care, and hence that outcome was not further analysed.

For intoxications alone, there were no significant pre-post differences for all eligible patients [*t*_±0.5_(32) = −0.215, *p* = .831; *t*_±1_(27) = −0.596, *p* = .556; *t*_±2_(21) = 1.133, *p* = .270], nor for patients with ongoing PIBA-contracts only [*t*_±0.5_(32) = −0.215, *p* = .831; *t*_±1_(22) = −1.679, *p* = .107; *t*_±2_(11) = 0.920, *p* = .377]. For patients with PIBA-contracts spanning over several years, the average number of contacts with the medical care due to intoxications were numerically stable over time. A sensitivity-analysis, excluding one patient who alone was responsible for 35 % of all contacts in the ± 2-year interval (all available data), did not reveal any significant effects (intention-to-treat: [*t*_±0.5_(31) = −1.161, *p* = .255; *t*_±1_(26) = −0.596, *p* = .556; *t*_±2_(20) = 0.627, *p* = .538], in-treatment: [*t*_±0.5_(31) = −1.161, *p* = .255; *t*_±1_(21) = −1.682, *p* = .107; *t*_±2_(10) = 0.247, *p* = .810]).

For self-harm, there was no significant pre-post differences for all eligible patients [*t*_±0.5_(32) = 0.205, *p* = .839; *t*_±1_(27) = −0.542, *p* = .592; *t*_±2_(21) = −1.670, *p* = .110], nor for patients with ongoing PIBA-contracts only [*t*_±0.5_(32) = 0.205, *p* = .839; *t*_±1_(22) = −1.105, *p* = .281; *t*_±2_(11) = −0.914, *p* = .380]. Two patients were responsible for nearly 80 % (57 % and 22 %, respectively) of the total number of contacts with medical care due to self-harm in the ± 2-year interval (all available data).

## Discussion

The main findings of this study were that PIBA-initiation coincided with a decrease in inpatient care under voluntary admission, as well as with a decrease in contacts with the psychiatric emergency department. PIBA did, however, not coincide with a change in self-inflicted injuries or in the number of days in compulsory care. Since PIBA is a comparatively low-cost intervention, and since PIBA is generally viewed favourably by patients (Strand & von Hausswolff-Juhlin, 2015; Varna *et al*., 2025), the results support a more widespread utilisation of PIBA.

Since this is a retrospective and uncontrolled study, the observed changes should not be interpreted as if they are necessarily causally related to the PIBA intervention. The analyses did not account for the effects of other interventions, in the analysed time interval, such as therapy or adjustment of prescription drugs. Moreover, since PIBA-contracts are more likely to be proposed and initiated when patients are undergoing some form of crisis or in a period with acute exacerbations, a lessened need for psychiatric care is to be expected during long-term follow-up (Westling *et al*., 2019; Nyttingnes & Ruud, 2020).

Early reports were optimistic about PIBA’s potential to reduce inpatient care (Strand & von Hausswolff-Juhlin, 2015). While some studies have reported a decrease (Skott *et al*., 2021), the majority of controlled studies have found no reduction (Sigrunarson *et al*., 2017; Westling *et al*., 2019; Eckerstrom *et al*., 2024) and some have even observed an increase in inpatient care (Thomsen *et al*., 2018). Moreover, it has been suggested that PIBA may decrease some measures, such as compulsory admissions specifically (Westling *et al*., 2019), or length of individual hospital stays (Eckerstrom *et al*., 2024). In our sample, total inpatient care decreased significantly for the 2-year pre-post analysis for all patients, but not in the pre-post analysis for patients that remained in the model only. What drives the significance is a decrease in voluntary admissions, and total inpatient care (excluding PIBA) decreased significantly in all pre-post analyses. The data hence suggest a shift from voluntary admissions to PIBA after initiation of contract, however due to the uncontrolled nature of the study no firm conclusions can be drawn. There were no significant effects on compulsory care, neither for all patients nor for patients with ongoing PIBA only.

Another suggested benefit of PIBA is a reduced number of contacts with the psychiatric emergency department (Strand & von Hausswolff-Juhlin, 2015; Johansson *et al*., 2023). In our cohort, there was a significant decrease in number of contacts with the psychiatric emergency department one year after, compared to one year before initiation of PIBA, but not for the other investigated intervals (Fig. 2). Following a peak in number of contacts prior to initiation of PIBA, the average number of contacts in the subsequent years was relatively low (less than two visits/year) and remained stable, on average, regardless of whether the patients had ongoing PIBA-contracts or not.

Intoxication was the most occurrent and prevalent type of self-inflicted injury in our cohort, while self-harm, apart from in two individuals, was a relatively rare event. Only one patient had strangulation events that resulted in contacts with medical care. There were no significant effects on self-inflicted injuries, which is in agreement with some (Thomsen *et al*., 2018), and in contrast to other (Westling *et al*., 2019) previous studies. This was true for self-inflicted injuries regardless of type, as well as for injuries stratified by type. Neither did sensitivity-analyses, excluding the three patients with the highest number of contacts, reveal any significant effects. This study used contacts with medical care as the outcome measure, while the previous positive report used data on non-suicidal self-injuries (NSSIs) (American Psychiatric Association, 2013) and attempted suicides obtained through the five self-harm behaviour groupings measure (5S-HM) (Liljedahl *et al*., 2023), which may be relevant to the difference in outcome. Unlike most other disadvantageous outcome measures in this study, intoxications remained, on average, stable over time for patients with ongoing PIBA-contracts.

There was little to no progression in the evaluated measures for patients with PIBA-contracts spanning over several years. This effect could potentially be due to confounding by severity, that is, that the patients who remain on PIBA-contracts are the one with the most persistent problems. Another possible explanation is that some patients may suffer from problems where PIBA might alleviate the symptoms, but does not, for these individuals, help them make meaningful progress in their lives. Although not part of the formal analysis, clinical familiarity with the patients and information obtained from their medical records during the manual review suggested that some individuals in our cohort had used PIBA extensively over several years, primarily to address issues related to loneliness. Another observation was that patients with expired contracts appeared, as a group, to have a lower overall need for psychiatric care, or were receiving care through means not captured by this study. Patient-reported outcomes, albeit not available in this study, could have offered more definitive insights into these questions.

The most important limitation of the present study is its retrospective and naturalistic nature. This, in combination with the data stemming from a single centre, means that the results should be interpreted with caution. No appropriate control group within our patient population was available for comparison with the investigated cohort. In this implementation of the model, PIBA admission was unavailable to patients during nighttime, which may have influenced the outcomes. Contacts with psychiatric or medical care outside of Kungälv Hospital were not assessed, and though such events were estimated to be relatively few, the study did not have 100 % event coverage. In the same vein, only self-harm events that resulted in contact with medical care were included. While not a limitation, the study cohort included relatively few patients with schizophrenia, and more female patients, compared to similar studies in mixed populations (Moljord *et al*., 2016; Thomsen *et al*., 2018; Skott *et al*., 2021). This should be taken into consideration when contrasting the present results to those from similar studies.

The study spanned over a relatively long time, following some patients up to 6 years after inclusion in the PIBA model. To date there are relatively few studies that evaluate long-term effects of PIBA (Nyttingnes & Ruud, 2020), though more are likely to be seen in the upcoming years as the intervention matures. This study also demonstrated the feasibility to evaluate PIBA through relevant measures readily collected from patients’ medical records, some of which cannot be obtained through national care registers (Thomsen *et al*., 2018; Eckerstrom *et al*., 2024). PIBA is widely implemented, and we encourage more caregivers that offer PIBA to perform similar evaluations to enable future high power meta-analyses.

## Conclusion

This research project describes the implementation of PIBA at Kungälv Hospital and demonstrates the feasibility to evaluate PIBA through measures readily collected from patients’ medical records. There was considerable inter-individual variability regarding PIBA utilisation and self-inflicted injuries. While PIBA-initiation coincided with a decrease in total inpatient care, the effect was numerically similar for patients independent on if they remained in the model or not and may thus, at least partly, be due to regression toward the mean. Looking at both the intention-to-treat and the in-treatment population, data were consistent with a shift from voluntary to self-referred inpatient care, which is likely to be beneficial from a health-economic perspective. Results on utilisation of psychiatric emergency services were generally in line with those on inpatient care. There were no significant changes in compulsory care or self-inflicted injuries. While the retrospective and naturalistic nature of the study precludes any strong conclusions, our results are generally in line with the majority of previous research on PIBA.

## Supporting information

Hagsäter et al. supplementary material 1 Hagsäter et al. supplementary material

Hagsäter et al. supplementary material 2 Hagsäter et al. supplementary material

## Supplementary material

The supplementary material for this article can be found at https://doi.org/10.1017/neu.2025.10031
