# Supplementary figures and images for: Patient-initiated brief admission: a single site eight-year retrospective cohort study
Source: Acta Neuropsychiatr. 2025 Aug 26;37:e80. doi: 10.1017/neu.2025.10031 (PMC13130303; doi:10.1017/neu.2025.10031)

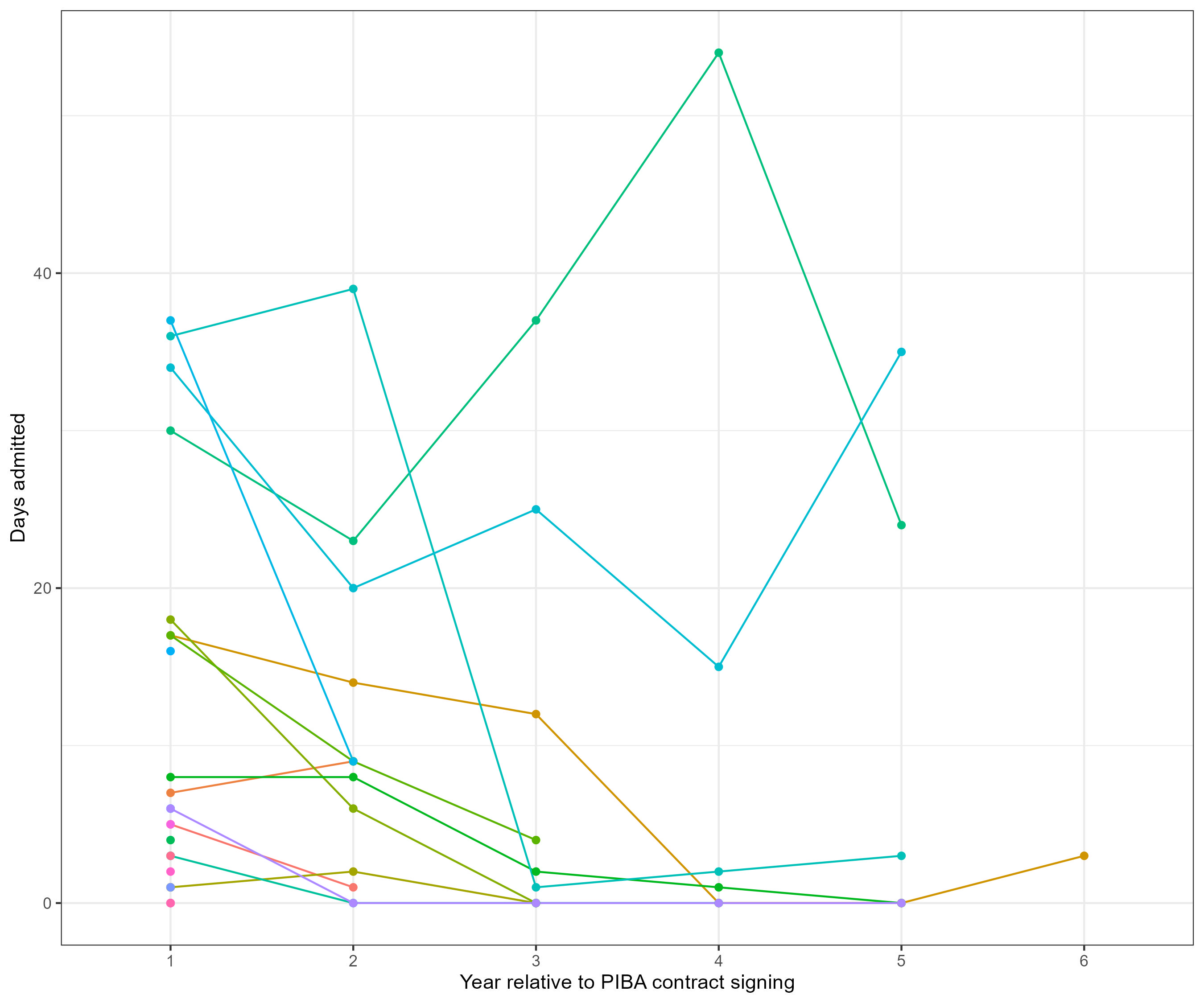

Supplement: Hagsäter et al. supplementary material 1 — Hagsäter et al. supplementary material [file S0924270825100318sup001.zip › 250530_SF1.jpg]

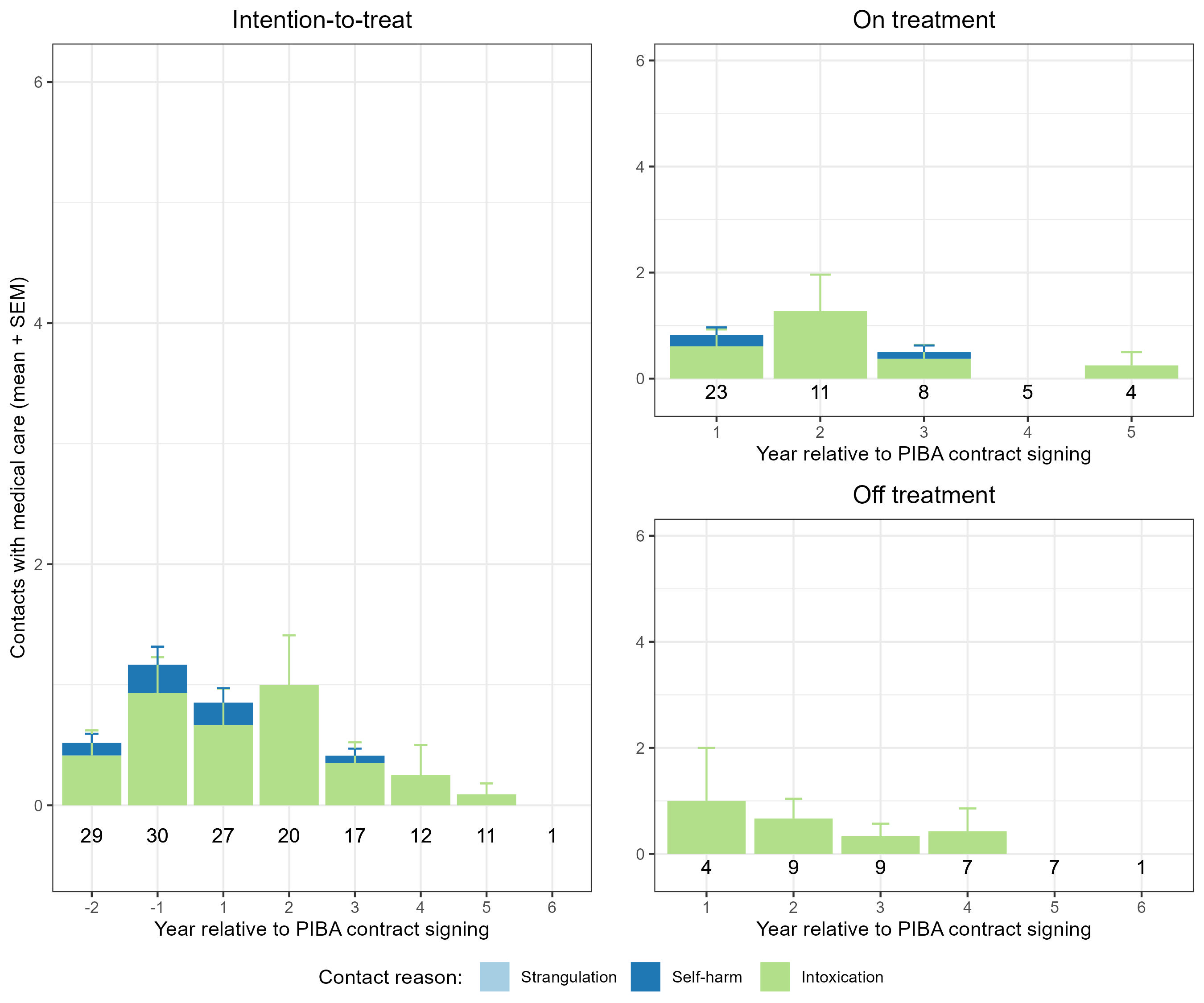

Supplement: Hagsäter et al. supplementary material 2 — Hagsäter et al. supplementary material [file S0924270825100318sup002.zip › 250530_SF2.jpg]
